# JGMS EDITOR’S CHOICE ARTICLES OF 2023: RISK AND RECOVERY OF FUNCTIONAL DECLINE AND FRAILTY

**DOI:** 10.1093/geroni/igae098.0653

**Published:** 2024-12-31

**Authors:** Lewis Lipsitz, Dae Kim

**Affiliations:** Chair; Discussant

## Abstract

This symposium will present four 2023 “Editor’s Choice” articles from the Journal of Gerontology Medical Sciences that focus on the risk and recovery of functional decline and frailty. Courtney Millar and her colleagues, in their article “Association of Proinflammatory Diet With Frailty Onset Among Adults With and Without Depressive Symptoms: Results From the Framingham Offspring Study” examine the association between an inflammatory diet and increased odds of frailty in those with depressive symptoms. Nicholas Rowan and his coauthors, in “The Association of Peripheral and Central Olfaction With Frailty in Older Adults” examine olfactory impairment, specifically olfactory identification and sensitivity, as a potential biomarker and risk factor for frailty. “Supplementing Glycine and N-Acetylcysteine (GlyNAC) in Older Adults Improves Glutathione Deficiency, Oxidative Stress, Mitochondrial Dysfunction, Inflammation, Physical Function, and Aging Hallmarks: A Randomized Clinical Trial”, written by Sekhar Rajagopal and colleagues, studies the effects of GlyNAC (combination of glycine and N-acetylcysteine [NAC]) supplementation on hallmarks of aging in older adults. Theresa Mau and coauthors, in their article, “Mitochondrial Energetics in Skeletal Muscle Are Associated With Leg Power and Cardiorespiratory Fitness in the Study of Muscle, Mobility and Aging,” examine the associations between mitochondrial energetics and functional outcomes, including leg power and cardiorespiratory fitness. Our discussant, Roger Fielding, will highlight commonalities and lessons learned from these studies.

